# Hydrocephalus as a consistent predictor of in-hospital mortality in tuberculous meningitis: no age-specific effect modification in a lifespan cohort

**DOI:** 10.3389/fneur.2026.1806668

**Published:** 2026-07-16

**Authors:** Qiong Wu, Xiangzhi Xiao, Huashan Zhou, Yan Ouyang, Sufen Chen, Jue Hu, Yanhua Zhou, Wengao Zeng

**Affiliations:** 1 Department of Neurosurgery, The Affiliated Changsha Central Hospital, Hengyang Medical School, University of South China, Changsha, Hunan, China; 2 Department of Internal Medicine, The Second Hospital of Changsha (West Branch of Changsha Hospital for Maternal & Child HealthCare), Changsha, Hunan, China; 3 Department of Pathology, The Second Hospital of Changsha (West Branch of Changsha Hospital for Maternal & Child HealthCare), Changsha, Hunan, China; 4 Department of Neurology, The Affiliated Changsha Central Hospital, Hengyang Medical School, University of South China, Changsha, Hunan, China; 5 Department of Otolaryngology, The Second Hospital of Changsha (West Branch of Changsha Hospital for Maternal & Child HealthCare), Changsha, Hunan, China

**Keywords:** age effect modification, hydrocephalus, in-hospital mortality, restricted cubic splines, tuberculous meningitis

## Abstract

**Background:**

Tuberculous meningitis (TBM) remains the most lethal manifestation of extrapulmonary tuberculosis, with persistently high in-hospital mortality despite advances in antimicrobial therapy. Hydrocephalus is a frequent and devastating complication of TBM. However, whether its prognostic impact on mortality is consistent across age groups—and whether age modifies this association—remains unresolved due to the lack of lifespan-spanning evidence.

**Methods:**

We conducted a single-center retrospective cohort study including non-HIV TBM patients hospitalized between October 2013 and January 2024. To address potential multicollinearity and reduce dimensionality, least absolute shrinkage and selection operator regression was applied for variable selection. This was followed by Firth's penalized logistic regression to estimate the adjusted associations with in-hospital mortality, ensuring robust statistical adjustment for confounding variables. We examined age-related effect modification by incorporating categorical and continuous interaction terms, performing stratified analyses, and applying restricted cubic splines modeling. Model performance was evaluated using discrimination, calibration, and decision curve analysis, with internal validation by bootstrap resampling.

**Results:**

Among 1,574 patients, the in-hospital mortality rate was 11.9%. Hydrocephalus was independently associated with increased mortality risk in the overall cohort, and this effect was broadly similar in children and adults. No statistically significant interaction between hydrocephalus and age was detected in categorical, continuous, or spline-based analyses. Predicted mortality increased with older age in both hydrocephalus groups, particularly in older patients, but the relative adverse effect of hydrocephalus remained broadly parallel across age. The prediction model showed excellent discrimination and good calibration, with stable net clinical benefit across a wide range of risk thresholds.

**Conclusions:**

Hydrocephalus is a strong predictor of in-hospital mortality in TBM. We found no statistically clear evidence of effect modification by age across the lifespan; however, this finding should be interpreted cautiously because the pediatric subgroup was relatively small and its confidence intervals were wider. These findings support early detection and timely management of hydrocephalus in patients of all ages.

## Introduction

Tuberculosis remains a leading infectious cause of death worldwide, and its most devastating manifestation is tuberculous meningitis (TBM) (1, 2). Despite advances in antituberculous therapy and supportive care, in-hospital mortality continues to range from 10% to 30% in contemporary cohorts, driven largely by delayed diagnosis and severe neurological complications (2, 3).

Hydrocephalus is one of the most common and feared complications of TBM (3, 4), arising from inflammatory obstruction of cerebrospinal fluid pathways and impaired absorption (5, 6). The resulting intracranial hypertension can rapidly cause cerebral hypoperfusion, ischemic injury, brainstem compression, and death (5, 7). Consequently, hydrocephalus has long been recognized as a key adverse prognostic factor (8, 9).

However, existing evidence on its prognostic importance is fragmented. Pediatric studies often report crude associations (10–14), whereas adult studies more consistently identify hydrocephalus as an independent predictor after multivariable adjustment (15, 16). Critically, these studies are almost invariably restricted to either children or adults, precluding direct comparison across the lifespan. No study to date has formally tested for age-related effect modification within a single unified cohort spanning children and adults.

We therefore conducted a large, lifespan-spanning retrospective cohort study of non-HIV TBM patients to determine (1) whether hydrocephalus is independently associated with in-hospital mortality, and (2) whether this association is modified by age.

## Materials and methods

### Study population

We conducted a single-center, retrospective observational cohort study of patients diagnosed with tuberculous meningitis at Changsha Central Hospital between October 1, 2013, and January 1, 2024. Eligible patients were identified from the hospital electronic medical record system according to pre-defined inclusion and exclusion criteria. This study was designed and reported in accordance with the Strengthening the Reporting of Observational Studies in Epidemiology (STROBE) guidelines (17). TBM was diagnosed according to internationally accepted consensus criteria, integrating clinical features, cerebrospinal fluid characteristics, neuroimaging findings, and microbiological evidence, as previously described (18). Exclusion criteria included (1) documented HIV co-infection; (2) concomitant intracranial infections, including bacterial or fungal meningitis; and (3) excessive missingness (>30%) in key covariates required for multivariable modeling. Patients were classified into pediatric ( ≤ 18 years; *n* = 211) and adult (>18 years; *n* = 1,363) groups based on age at admission. The index date was defined as the date of first hospitalization for TBM, and follow-up was restricted to the inpatient period. The study was approved by the Ethics Committee of Changsha Central Hospital (Approval No. 20250122). Given the retrospective design and the use of anonymized data, the requirement for written informed consent was waived by the Ethics Committee.

### Data collection and variable definitions

Demographic characteristics, clinical manifestations, laboratory findings (blood and CSF), and neuroimaging data were systematically extracted from electronic medical records by two independent investigators. Discrepancies were resolved through joint review and consensus, and data completeness and plausibility were checked before analysis.

To minimize potential reverse causation, continuous variables were recorded using values obtained at hospital admission or at the earliest time point prior to outcome occurrence.

## Outcome

The primary outcome was in-hospital mortality, defined as death from any cause during the index TBM hospitalization.

### Exposure

Hydrocephalus was defined as ventriculomegaly on cranial CT or MRI, quantified by an Evans' index (ratio of maximal frontal horn width to maximal intracranial width) greater than 0.3, as reported by attending neuroradiologists (19). Clinical relevance was assessed by the presence of signs of raised intracranial pressure (e.g., impaired consciousness) and the potential need for cerebrospinal fluid diversion procedures, such as external ventricular drainage or ventriculoperitoneal shunting (20). Hydrocephalus status was determined at baseline, using neuroimaging performed on admission or the earliest available scan, to ensure temporal precedence relative to the outcome. This definition aligns with established radiological criteria in tuberculous meningitis studies and World Federation of Neurosurgical Societies guidelines.

### Covariates

Candidate covariates included demographic factors, comorbidities, laboratory parameters, and other clinically or biologically plausible predictors of TBM prognosis identified in prior literature.

**Drug-induced liver injury (DILI)** was defined as liver biochemical abnormalities occurring after hospital admission that were temporally associated with anti-tuberculous therapy, in the absence of alternative etiologies. Specifically, DILI was diagnosed if any of the following criteria were met during hospitalization: (1) alanine aminotransferase (ALT) ≥ 3 × the upper limit of normal (ULN) with liver-related symptoms; (2) ALT ≥ 5 × ULN irrespective of symptoms; (3) alkaline phosphatase (ALP) ≥ 2 × ULN, particularly when accompanied by elevated γ-glutamyl transferase (GGT); or (4) total bilirubin ≥ 2 × ULN in combination with elevated ALT or ALP, with other causes of liver injury (e.g., viral hepatitis, autoimmune hepatitis, ischemic hepatitis) reasonably excluded (21). Only liver injury events occurring after initiation of anti-tuberculous therapy during the index hospitalization were considered, and patients with documented chronic liver disease at baseline were classified separately (22, 23).

**Altered consciousness** was defined as a Glasgow Coma Scale (GCS) score < 15 at admission (24).

**Hyponatremia** was defined as a serum sodium concentration < 135 mmol/L measured within 24 h of admission (25).

**Anemia** was defined according to World Health Organization criteria, using hemoglobin thresholds of < 130 g/L in adult men, < 120 g/L in adult women, and age-adjusted thresholds in children (26).

**Cerebral infarction** was defined as radiologically confirmed acute or subacute ischemic lesions on CT or MRI consistent with vascular territory infarction (27).

**Immunodeficiency:** This study focused on secondary immunodeficiency, defined as an acquired immune system dysfunction leading to increased susceptibility to infections. HIV-positive patients were excluded. Secondary immunodeficiency was characterized by (1) laboratory evidence of reduced lymphocyte subsets, including CD4+ T-cell counts < 200 cells/μl or other abnormal B-cell counts, and (2) immunodeficiency induced by the use of corticosteroids or other immunosuppressive agents (e.g., glucocorticoids, cyclosporine). Classification and diagnostic criteria were grounded in international standards for immunodeficiency disorders, including World Health Organization (WHO) conceptual classifications of immune dysfunction, and referenced guidance from the National Institute of Allergy and Infectious Diseases (NIAID) on immune system disorders and immune suppression contexts (28).

**Renal failure** was defined according to KDIGO-based criteria for acute kidney injury, including a serum creatinine increase ≥1.5 times baseline or urine output < 0.5 ml/kg/h for 6 h (29).

Brain MRI was performed using a 1.5T or 3.0T scanner with sequences including T1-weighted, T2-weighted, FLAIR, and contrast-enhanced imaging. Images were interpreted blindly by two senior radiologists to ensure objectivity, with classifications based on TBM-specific radiological patterns. Brain MRI findings were categorized according to commonly reported TBM neuroimaging patterns, including normal findings, parenchymal lesions, meningeal enhancement, complicated lesions, and spinal involvement.

Normal: Normal neuroimaging was defined as the absence of signal abnormalities or structural alterations.

Parenchymal lesions: Focal or diffuse abnormal signals in the brain parenchyma, such as infarcts, tuberculomas, or encephalitis. Typical features include T2/FLAIR hyperintense areas, ring-enhancing lesions on post-contrast T1 (characteristic of tuberculomas), or restricted diffusion on DWI/ADC (indicative of acute infarction).

Meningeal enhancement: Abnormal meningeal enhancement post-contrast, indicating inflammation or tumor infiltration. Typically involves the basal cisterns and fissures (e.g., sylvian fissures, tentorium), appearing as linear or nodular hyperintensity on post-contrast T1, often accompanied by leptomeningitis.

Complicated: Accompanied by multiple complications, such as hemorrhage, edema, infarction, or hydrocephalus. Imaging features include multiple infarcts (e.g., periventricular infarcts), brain edema (T2/FLAIR hyperintensity), hemorrhage (hypointensity on GRE sequences), and ventricular enlargement.

Spinal involvement: Abnormal spinal cord signals or enhancement, indicating spinal extension. Features include spinal cord T2 hyperintensity, post-contrast enhancement, or subarachnoid adhesions.

## Statistical analysis

### Descriptive analysis

Continuous variables were summarized as medians (interquartile ranges), and categorical variables as counts (percentages). Differences between survivors and non-survivors were assessed using the Wilcoxon rank-sum test or Fisher's exact test, as appropriate.

### Model development and variable selection

To reduce dimensionality and mitigate multicollinearity, least absolute shrinkage and selection operator (LASSO) regression was first applied for preliminary variable selection (30). Selected variables were subsequently entered into multivariable logistic regression models. Given the low event rates in certain subgroups, Firth's penalized likelihood logistic regression was employed to reduce small-sample bias and address complete or quasi-complete separation (31).

### Assessment of the effect of hydrocephalus

Adjusted odds ratios (aORs) and 95% confidence intervals were estimated for the association between hydrocephalus and in-hospital mortality in the overall cohort, as well as in pediatric and adult subgroups.

### Age effect-modification analysis

1. To comprehensively evaluate whether age modified the association between hydrocephalus and mortality, several complementary strategies were employed:
2. Categorical interaction analyses: inclusion of hydrocephalus, age group (children vs. adults), and their interaction term in multivariable models;
3. Continuous interaction analyses: modeling age as a continuous variable and testing the hydrocephalus × age interaction;
4. Stratified analyses: estimation and comparison of aORs within pediatric and adult strata;
5. Non-linear interaction analyses: restricted cubic splines with four knots were used to characterize potential non-linear relationships between age and mortality risk (32).

### Model performance and internal validation

Model performance was evaluated according to the Transparent Reporting of a multivariable prediction model for Individual Prognosis or Diagnosis (TRIPOD) guidelines, assessing discrimination (AUC), calibration (calibration plots), and clinical utility (decision curve analysis) (33). Internal validation was performed using bootstrap resampling (200 iterations). The number of resamples was selected to balance computational feasibility and estimation stability, with consistent performance observed across resamples. Patients with excessive missingness (>30%) in key covariates were excluded. The primary analysis was conducted using complete cases for variables included in each model. All analyses were conducted using R software (version 4.5.2). Two-sided *P*-values < 0.05 were considered statistically significant.

## Results

### Patient characteristics

**Figure 1:**
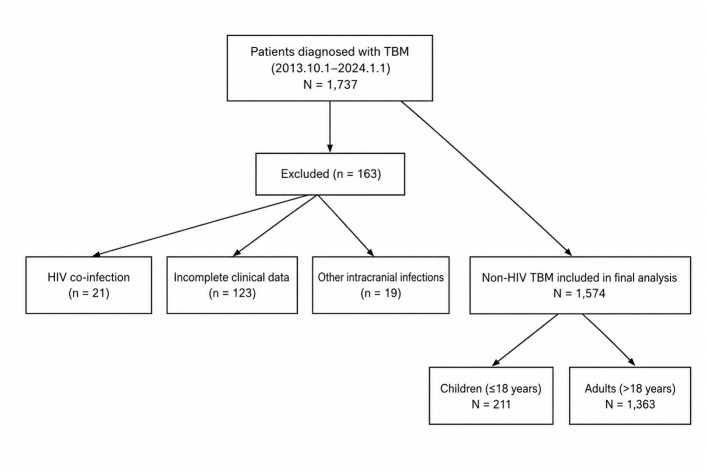
Flow diagram of patient selection. Of 1,737 patients diagnosed with tuberculous meningitis between October 2013 and January 2024, 1,574 non-HIV patients were included after excluding those with HIV co-infection (*n* = 21), incomplete data (*n* = 123), and concomitant intracranial infections (*n* = 19). The cohort comprised 211 children ( ≤ 18 years) and 1,363 adults.

Among 1,737 patients diagnosed with TBM during the study period, 1,574 non-HIV patients were included after exclusions (Figure 1). Of these, 211 (13.4%) were children ( ≤ 18 years) and 1,363 (86.6%) were adults. Overall, in-hospital mortality was 11.9% (187/1,574).

In the full cohort, non-survivors were significantly more likely to present with hydrocephalus, immunodeficiency, drug-induced liver injury, hyponatremia, and renal failure compared with survivors (all *P* < 0.05). Detailed clinical characteristics stratified by outcome are provided in Table 1.

**Table 1:** Patient characteristics of TBM patients stratified by outcome (survival vs. non-survival).

Variable	Overall *N* = 1,574	Survival *N* = 1,387	Non-survival *N* = 187	*P*-value
Demographics				
Age (years), median (IQR)	44.00 (25.00, 59.00)	44.00 (25.00, 59.00)	45.00 (28.00, 62.00)	0.055
Female, *n* (%)	1,035 (66%)	906 (65%)	129 (69%)	0.400
Comorbidities and clinical features				
Pulmonary tuberculosis type, *n* (%)				0.600
No pulmonary tuberculosis	347 (22%)	310 (22%)	37 (20%)	
Hematogenous dissemination	490 (31%)	422 (30%)	68 (36%)	
Primary pulmonary tuberculosis	53 (3.4%)	48 (3.5%)	5 (2.7%)	
Secondary pulmonary tuberculosis	679 (43%)	602 (43%)	77 (41%)	
Tuberculous pleurisy	5 (0.3%)	5 (0.4%)	0 (0%)	
DILI, *n* (%)	116 (7.4%)	18 (1.3%)	98 (52%)	**< 0.001**
Hydrocephalus, *n* (%)	119 (7.6%)	88 (6.3%)	31 (17%)	**< 0.001**
Cerebral hemorrhage, *n* (%)	16 (1.0%)	11 (0.8%)	5 (2.7%)	0.033
Immunodeficiency, *n* (%)	452 (29%)	374 (27%)	78 (42%)	< 0.001
Cerebral infarction, *n* (%)	265 (17%)	228 (16%)	37 (20%)	0.3
Epilepsy, *n* (%)	86 (5.5%)	61 (4.4%)	25 (13%)	< 0.001
History of tuberculosis contact, *n* (%)	318 (20%)	284 (20%)	34 (18%)	0.5
Renal failure, *n* (%)	412 (26%)	334 (24%)	78 (42%)	< 0.001
Laboratory findings				
Fibrinogen (g/L), median (IQR)	4.60 (2.83, 4.60)	4.60 (2.84, 4.60)	4.60 (2.83, 4.60)	0.2
Serum uric acid (μmol/L), median (IQR)	370.50 (250.00, 524.00)	374.70 (250.00, 529.70)	296.00 (196.00, 515.00)	0.023
Hyponatremia	442 (28%)	367 (26%)	75 (40%)	< 0.001
Serum creatinine (μmol/L), median (IQR)	48.00 (36.00, 65.00)	47.00 (36.00, 63.00)	54.40 (36.00, 78.00)	< 0.001
CSF culture	30 (1.9%)	30 (2.2%)	0 (0%)	0.041
CSF white blood cell count ( × 10^6^/L), median (IQR)	10.00 (10.00, 60.00)	10.00 (10.00, 51.00)	11.00 (10.00, 100.00)	0.031
Proportion of mononuclear cells in CSF, median (IQR)	0.68 (0.50, 0.69)	0.68 (0.50, 0.69)	0.67 (0.50, 0.69)	0.044
CSF chloride (mmol/L), median (IQR)	124.20 (115.00, 125.00)	125.00 (116.00, 125.00)	121.00 (113.00, 125.00)	0.016
CSF glucose (mmol/L), median (IQR)	2.20 (2.20, 3.21)	2.20 (2.20, 3.21)	2.20 (1.93, 3.21)	0.2
CSF protein (g/L), median (IQR)	0.90 (0.59, 1.37)	0.90 (0.58, 1.32)	0.91 (0.60, 1.84)	0.013
CSF ADA (U/L), median (IQR)	2.43 (2.43, 3.00)	2.43 (2.43, 2.80)	2.43 (2.43, 5.00)	0.038
CSF Mycobacterium tuberculosis DNA detected, *n* (%)	45 (2.9%)	40 (2.9%)	5 (2.7%)	>0.9
Platelet count ( × 10^9^/L), median (IQR)	217.00 (154.00, 275.00)	217.00 (158.00, 275.00)	216.00 (125.00, 272.00)	0.052
ESR (mm/h), median (IQR)	20.00 (19.00, 39.00)	20.00 (18.00, 37.00)	20.00 (20.00, 46.00)	0.002
Hemoglobin (g/L), median (IQR)	114.15 (105.00, 126.00)	115.00 (105.00, 126.00)	110.00 (101.70, 119.00)	< 0.001
Indirect bilirubin (μmol/L), median (IQR)	5.50 (3.30, 5.90)	5.50 (3.30, 5.86)	5.50 (3.42, 6.36)	0.5
PCT (ng/ml), median (IQR)	0.04 (0.03, 0.09)	0.04 (0.03, 0.08)	0.05 (0.03, 0.28)	< 0.001
log_PCT, median (IQR)	−3.00 (-3.22,−2.30)	−3.00 (-3.22,−2.41)	−2.81 (-3.22,−1.24)	< 0.001
Albumin (g/L), median (IQR)	35.00 (32.00, 38.50)	35.00 (32.50, 38.80)	34.30 (29.00, 37.00)	< 0.001
ICP (mmH_2_O), median (IQR)	150.00 (120.00, 190.00)	150.00 (125.00, 190.00)	150.00 (120.00, 220.00)	0.2
Drug-resistant tuberculosis, *n* (%)	98 (6.2%)	83 (6.0%)	15 (8.0%)	0.3
PPD positivity	131 (8.3%)	114 (8.2%)	17 (9.1%)	0.7
Diagnostic tests				
CSF tuberculosis antibody positive, *n* (%)	195 (12%)	165 (12%)	30 (16%)	0.12
CSF acid-fast bacilli smear positive, *n* (%)	7 (0.4%)	6 (0.4%)	1 (0.5%)	0.6
Treatment				
Corticosteroid, *n* (%)	1,535 (98%)	1,351 (97%)	184 (98%)	0.6
Levofloxacin, *n* (%)	859 (55%)	758 (55%)	101 (54%)	0.9
Linezolid, *n* (%)	996 (63%)	884 (64%)	112 (60%)	0.3
Aspirin use, *n* (%)	844 (54%)	737 (53%)	107 (57%)	0.3
Brain MRI findings, *n* (%)				
Normal	226 (14%)	205 (15%)	21 (11%)	0.20
Parenchymal lesions	1,068 (68%)	939 (68%)	129 (69%)	
Meningeal enhancement	160 (10%)	140 (10%)	20 (11%)	
Complicated	81 (5.1%)	66 (4.8%)	15 (8.0%)	
Spinal involvement	39 (2.5%)	37 (2.7%)	2 (1.1%)	
TBM diagnostic category, *n* (%)				
Definite	553 (35%)	493 (36%)	60 (32%)	0.20
Probable	953 (61%)	830 (60%)	123 (66%)	
Possible	68 (4.3%)	64 (4.6%)	4 (2.1%)	

This table summarizes the demographic, clinical, laboratory, diagnostic, and treatment characteristics of 1,574 non-HIV TBM patients, stratified by outcome.

Continuous variables are presented as median (IQR), and categorical variables as *n* (%).

*P*-values were derived from the Wilcoxon rank-sum test for continuous variables and Fisher's exact test for categorical variables. Some variables, such as DILI, represent in-hospital complications rather than pre-admission features.

UA, uric acid; ALB, albumin; DILI, drug-induced liver injury; CSF, cerebrospinal fluid; AFB, acid-fast bacilli; PPD, purified protein derivative; PCT, procalcitonin; ESR, erythrocyte sedimentation rate; ICP, intracranial pressure; TBM, tuberculous meningitis; IQR, interquartile range.

Among non-survivors, adults had a higher prevalence of comorbidities such as immunodeficiency and renal failure compared with children (*P* < 0.05), whereas the prevalence of hydrocephalus did not differ significantly between age groups. Clinical characteristics of non-survivors stratified by age are summarized in Table 2.

**Table 2:** Comparison of demographic, clinical, and laboratory characteristics among in-hospital non-survivors with non-HIV TBM by age group.

Variable	Overall *N* = 187	Child *N* = 22	Adult *N* = 165	*P*-value
Demographics				
Age (years), median (IQR)	45.00 (28.00, 62.00)	10.00 (2.00, 15.00)	51.00 (33.00, 66.00)	< 0.001
Female, *n* (%)	129 (69%)	11 (50%)	118 (72%)	0.051
Comorbidities and clinical features				
Pulmonary tuberculosis type, *n* (%)				< 0.001
No pulmonary tuberculosis	37 (20%)	6 (27%)	31 (19%)	
Hematogenous dissemination	68 (36%)	7 (32%)	61 (37%)	
Primary pulmonary tuberculosis	5 (2.7%)	5 (23%)	0 (0%)	
Secondary pulmonary tuberculosis	77 (41%)	4 (18%)	73 (44%)	
Tuberculous pleurisy	0 (0%)	0 (0%)	0 (0%)	
DILI, *n* (%)	98 (52%)	11 (50%)	87 (53%)	0.8
Hydrocephalus, *n* (%)	31 (17%)	6 (27%)	25 (15%)	0.2
Cerebral hemorrhage, *n* (%)	5 (2.7%)	0 (0%)	5 (3.0%)	>0.9
Immunodeficiency, *n* (%)	78 (42%)	3 (14%)	75 (45%)	0.005
Cerebral infarction, *n* (%)	37 (20%)	2 (9.1%)	35 (21%)	0.3
Epilepsy, *n* (%)	25 (13%)	4 (18%)	21 (13%)	0.5
History of tuberculosis contact, *n* (%)	34 (18%)	3 (14%)	31 (19%)	0.8
Renal failure, *n* (%)	78 (42%)	2 (9.1%)	76 (46%)	< 0.001
Laboratory findings				
Fibrinogen (g/L), median (IQR)	4.60 (2.83, 4.60)	4.60 (2.58, 4.60)	4.60 (2.83, 4.60)	0.7
Serum uric acid (μmol/L), median (IQR)	296.00 (196.00, 515.00)	250.00 (156.00, 475.50)	317.00 (206.00, 516.00)	0.070
Hyponatremia	75 (40%)	10 (45%)	65 (39%)	0.6
Serum creatinine (μmol/L), median (IQR)	54.40 (36.00, 78.00)	36.00 (36.00, 47.40)	57.00 (41.00, 81.00)	< 0.001
CSF culture	0 (0%)	0 (0%)	0 (0%)	>0.9
CSF white blood cell count ( × 106/L), median (IQR)	11.00 (10.00, 100.00)	10.00 (8.00, 80.00)	12.00 (10.00, 100.00)	0.5
Proportion of mononuclear cells in CSF, median (IQR)	0.67 (0.50, 0.69)	0.60 (0.50, 0.69)	0.67 (0.50, 0.69)	0.5
CSF chloride (mmol/L), median (IQR)	121.00 (113.00, 125.00)	118.45 (114.00, 125.00)	121.10 (113.00, 125.00)	>0.9
CSF glucose (mmol/L), median (IQR)	2.20 (1.93, 3.21)	2.20 (1.41, 3.07)	2.20 (1.98, 3.21)	0.5
CSF protein (g/L), median (IQR)	0.91 (0.60, 1.84)	0.85 (0.50, 1.26)	0.96 (0.66, 1.86)	0.13
CSF ADA (U/L), median (IQR)	2.43 (2.43, 5.00)	2.43 (2.43, 2.43)	2.43 (2.43, 5.00)	0.5
CSF Mycobacterium tuberculosis DNA detected, *n* (%)	5 (2.7%)	0 (0%)	5 (3.0%)	>0.9
Platelet count ( × 10?/L), median (IQR)	216.00 (125.00, 272.00)	339.00 (255.00, 469.00)	201.00 (125.00, 241.00)	< 0.001
ESR (mm/h), median (IQR)	20.00 (20.00, 46.00)	22.50 (18.00, 39.00)	20.00 (20.00, 46.00)	0.5
Hemoglobin (g/L), median (IQR)	110.00 (101.70, 119.00)	110.75 (102.20, 120.20)	110.00 (101.40, 119.00)	0.5
PCT (ng/ml), median (IQR)	0.05 (0.03, 0.28)	0.05 (0.03, 0.10)	0.05 (0.03, 0.28)	0.4
log_PCT, median (IQR)	−2.81 (-3.22,−1.24)	−2.90 (-3.22,−2.21)	−2.81 (-3.22,−1.24)	0.4
Albumin (g/L), median (IQR)	34.30 (29.00, 37.00)	35.00 (34.60, 39.20)	34.00 (28.90, 36.90)	0.039
ICP (mmH_2_O), median (IQR)	150.00 (120.00, 220.00)	192.50 (130.00, 250.00)	150.00 (120.00, 215.00)	0.2
Drug-resistant tuberculosis, *n* (%)	15 (8.0%)	0 (0%)	15 (9.1%)	0.2
PPD positivity	17 (9.1%)	9 (41%)	8 (4.8%)	< 0.001
Diagnostic tests				
CSF tuberculosis antibody positive, *n* (%)	30 (16%)	5 (23%)	25 (15%)	0.4
CSF acid-fast bacilli smear positive, *n* (%)	1 (0.5%)	0 (0%)	1 (0.6%)	>0.9
Treatment				
Corticosteroid, *n* (%)	184 (98%)	22 (100%)	162 (98%)	>0.9
Levofloxacin, *n* (%)	101 (54%)	12 (55%)	89 (54%)	>0.9
Linezolid, *n* (%)	112 (60%)	16 (73%)	96 (58%)	0.2
Aspirin use, *n* (%)	107 (57%)	11 (50%)	96 (58%)	0.5
**Brain MRI findings**, ***n*** **(%)**				0.80
Normal	21 (11%)	1 (4.5%)	20 (12%)	
Parenchymal lesions	129 (69%)	18 (82%)	111 (67%)	
Meningeal enhancement	20 (11%)	2 (9.1%)	18 (11%)	
Complicated	15 (8.0%)	1 (4.5%)	14 (8.5%)	
Spinal involvement	2 (1.1%)	0 (0%)	2 (1.2%)	
**TBM diagnostic category**, ***n*** **(%)**				0.40
Definite	60 (32%)	10 (45%)	50 (30%)	
Probable	123 (66%)	12 (55%)	111 (67%)	
Possible	4 (2.1%)	0 (0%)	4 (2.4%)	

This table summarizes the demographic, clinical, laboratory, diagnostic, and treatment characteristics of deceased TBM patients (*N* = 187), stratified by age group (children ≤ 18 years, *N* = 22; adults >18 years, *N* = 165).

Continuous variables are presented as median (IQR), and categorical variables as *n* (%). *P*-values were derived from the Wilcoxon rank-sum test for continuous variables and Fisher's exact test for categorical variables.

Significant differences (*p* < 0.05) are observed in variables such as age, pulmonary tuberculosis type, immunodeficiency, renal failure, serum creatinine, platelet count, PPD positivity, and albumin, indicating age-specific patterns in mortality-associated factors.

DILI, drug-induced liver injury; IQR, interquartile range; CSF, cerebrospinal fluid; PCT, procalcitonin; ESR, erythrocyte sedimentation rate; PPD, purified protein derivative; ICP, intracranial pressure; TBM, tuberculous meningitis.

### Model performance

**Figure 2:**
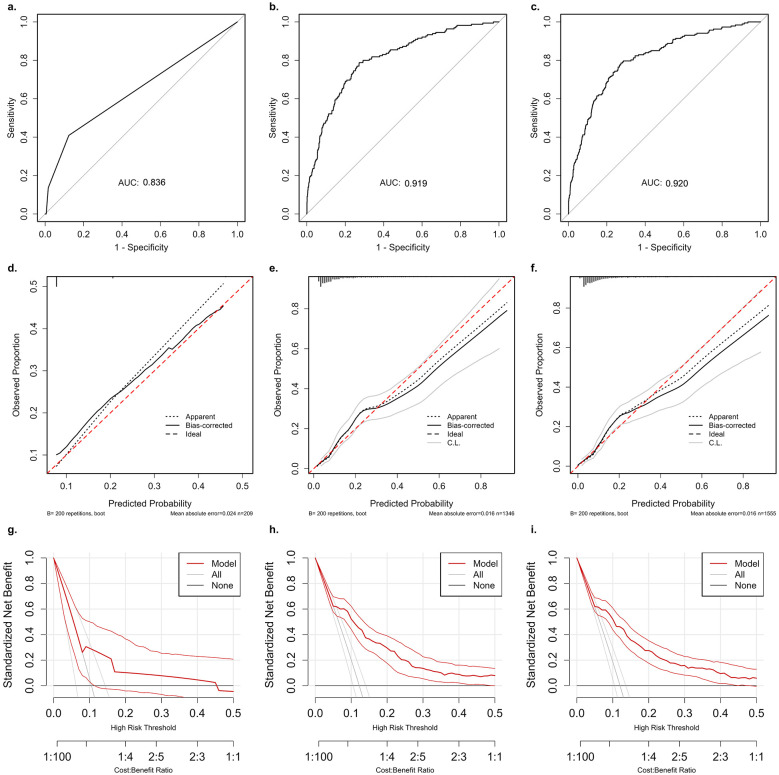
Performance of the prognostic model for in-hospital mortality in non-HIV TBM patients, stratified by age group. Panels **(a–c)** show receiver operating characteristic curves (AUC: 0.836 in children, 0.919 in adults, and 0.920 overall); panels **(d–f)** show calibration plots; and panels **(g–i)** show decision curve analyses. Models were developed using Firth-penalized logistic regression after LASSO variable selection, with bootstrap internal validation (200 iterations). AUC, area under the curve; DCA, decision curve analysis.

The final prediction model demonstrated excellent discrimination (AUC 0.920 overall, 0.919 adults, 0.836 children) and good calibration, with stable performance after bootstrap internal validation (Figure 2).

### Internal validation

After 200 bootstrap resamples, only minimal attenuation in discrimination was observed, with optimism-corrected AUC values closely approximating the apparent AUC. Bootstrap-corrected calibration curves showed no evidence of systematic over- or under-estimation of risk, indicating good internal stability and low overfitting (see Figure 2).

### Hydrocephalus as an independent predictor of in-hospital mortality

**Figure 3:**
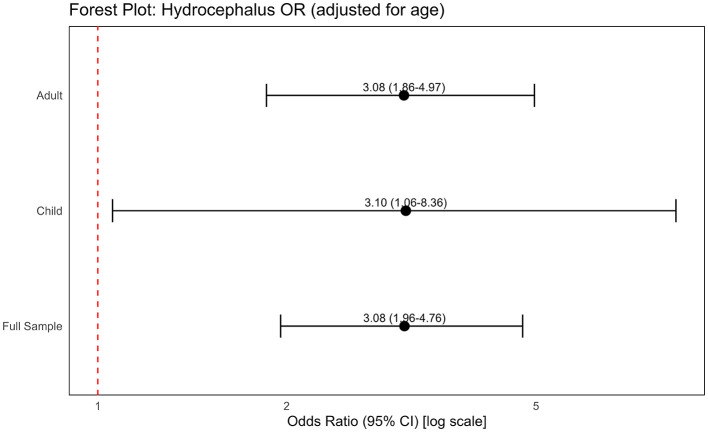
Forest plot of adjusted odds ratios for the association between hydrocephalus and in-hospital mortality in the overall cohort and age strata. Models were adjusted for confounders using Firth-penalized logistic regression after LASSO variable selection. Point estimates and 95% confidence intervals are shown on a log scale; the red line indicates no effect. Data from 1,574 patients.

Hydrocephalus was strongly associated with mortality after multivariable adjustment (aOR 3.08, 95% CI 1.96–4.76). Subgroup estimates were broadly similar: children aOR 3.10 (95% CI 1.06–8.36); adults aOR 3.08 (95% CI 1.86–4.97; Figure 3).

### Effect modification by age

**Figure 4:**
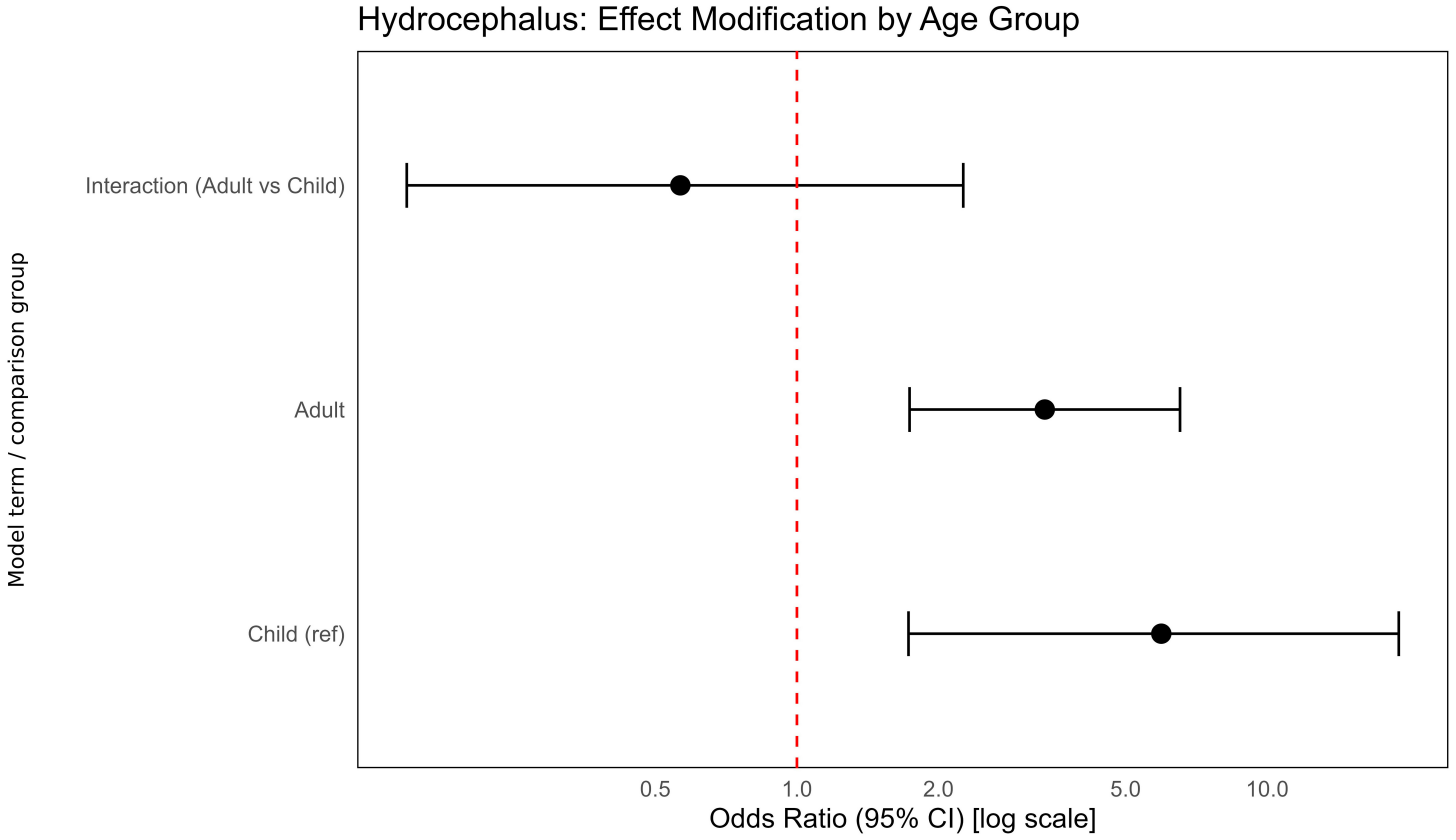
Forest plot showing age-group interaction analysis for the hydrocephalus-mortality association. Models were adjusted for confounders using Firth-penalized logistic regression after LASSO variable selection. Point estimates and 95% confidence intervals are shown on a log scale; the red line indicates no effect. The adult-vs.-child interaction term was not statistically significant (*P* = 0.402). Data from 1,574 patients.

**Figure 5:**
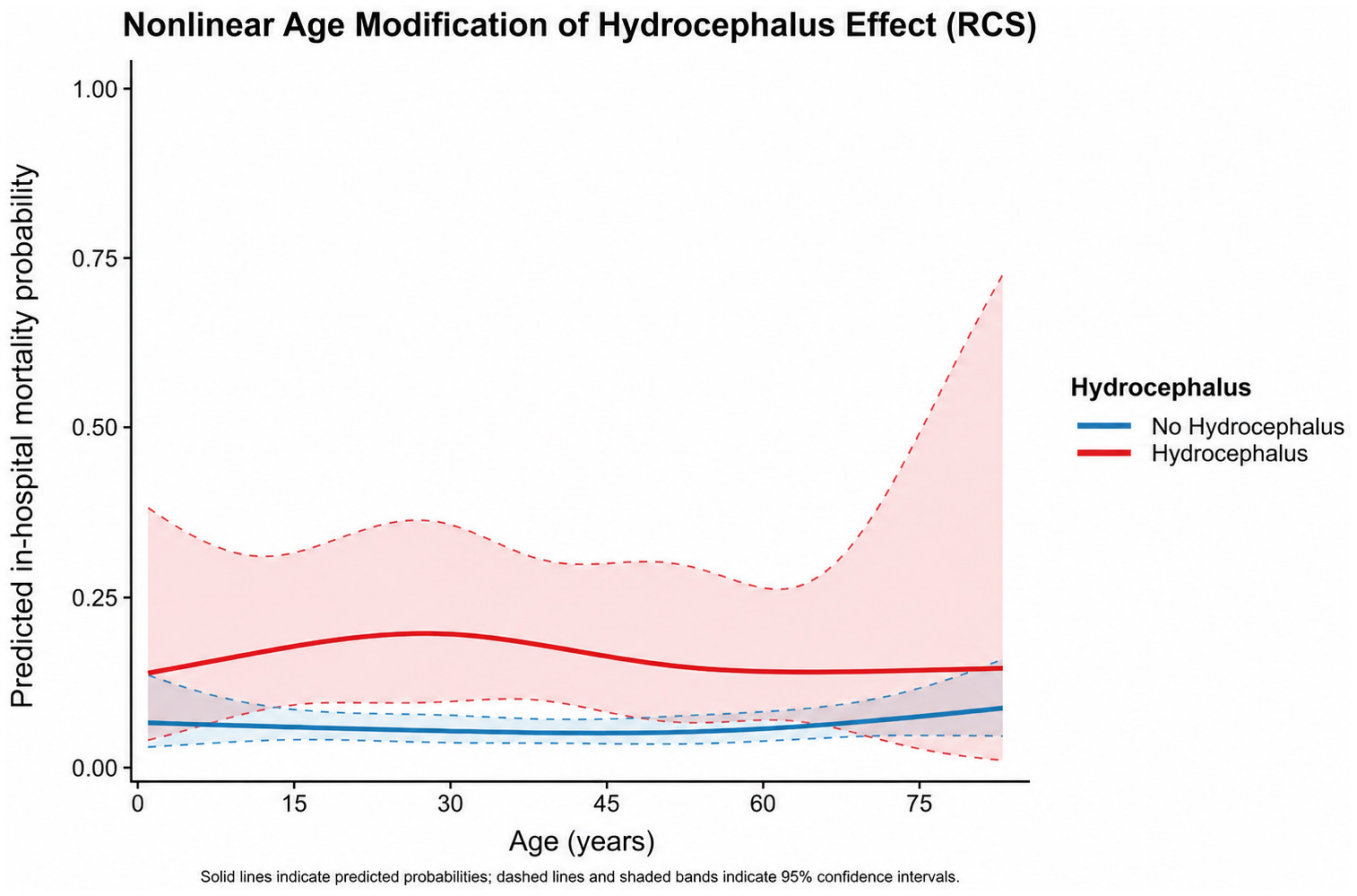
Restricted cubic spline analysis evaluating non-linear age-related modification of the hydrocephalus-mortality association in non-HIV tuberculous meningitis patients. Predicted in-hospital mortality probability by continuous age is shown according to hydrocephalus status. Blue indicates no hydrocephalus, and red indicates hydrocephalus. Shaded bands and dashed lines indicate the corresponding 95% confidence intervals, with colors matched to the curves. Predicted mortality increased with older age in both groups, particularly among older patients, but the relative adverse effect of hydrocephalus remained broadly parallel across age, with no significant non-linear interaction (*P* = 0.402). Abbreviations: RCS, restricted cubic spline; TBM, tuberculous meningitis.

No statistically clear evidence of age-related effect modification was observed. The interaction term between hydrocephalus and age group was non-significant (*P* = 0.402), as was the continuous age interaction (*P* > 0.6). Figure 4 presents the categorical age-group interaction analysis, showing overlapping effect estimates between children and adults and no statistically significant adult-vs.-child interaction. Restricted cubic spline analysis showed broadly parallel risk curves across age without significant non-linear interaction (*P* = 0.402; Figure 5). Although the interaction analyses did not show statistically clear evidence of age-related effect modification, the predicted absolute mortality probability increased with older age in both hydrocephalus groups. This finding indicates that age remains clinically relevant for absolute mortality risk stratification despite the absence of statistically clear effect modification.

## Discussion

In this large lifespan cohort of non-HIV TBM patients, hydrocephalus was a robust independent predictor of in-hospital mortality, with no statistically clear evidence of age-related effect modification. The adjusted odds ratio of approximately 3 was broadly similar in children and adults across multiple analytical approaches. Importantly, the absence of statistically significant interaction does not imply that age has no prognostic relevance. The spline analysis suggested an increase in absolute predicted mortality probability with older age in both hydrocephalus strata, indicating that age remains clinically relevant for absolute risk stratification even though it did not statistically modify the relative hydrocephalus-mortality association.

Our findings extend previous age-specific studies by directly comparing the prognostic impact of hydrocephalus across children and adults within a unified cohort. Although pediatric and adult studies have historically reported distinct risk profiles—with higher crude mortality in children (13, 34) and older age as a risk factor in adults (15)—our direct comparison of these two groups reveals that hydrocephalus has a broadly similar adverse impact across age groups on mortality. This broadly similar pattern may reflect a shared pathophysiological endpoint: sustained intracranial hypertension causing reduced cerebral perfusion, ischemic injury, and irreversible secondary brain damage that overrides age-related physiological differences (7, 35–37).

However, it is important to note that despite this broadly similar pattern, the wider confidence intervals in the pediatric subgroup (due to smaller sample size) mean that subtle effect modification cannot be entirely excluded. Further research with larger, multicenter studies is necessary to validate these findings across diverse populations.

Clinically, these results support a unified approach to hydrocephalus management across all age groups, with equal emphasis on early neuroimaging, close monitoring of intracranial pressure, and timely cerebrospinal fluid diversion when indicated. Prior literature has consistently reported a two- to threefold increased mortality risk with hydrocephalus (15, 38); our larger sample and rigorous confounder adjustment provide more precise estimates.

The predictive model incorporating hydrocephalus showed excellent discrimination (AUC 0.92), good calibration, and clinical utility on decision curve analysis, highlighting its potential role in risk stratification.

Study strengths include the large lifespan-spanning cohort, advanced variable selection and penalized regression to address separation, and comprehensive testing for effect modification using interaction terms and restricted cubic splines.

Despite the strengths of this study, several limitations must be acknowledged. First, as a single-center retrospective cohort, there is a potential for selection bias, which could affect the generalizability of the findings to other populations or healthcare settings. Additionally, the relatively small sample size of pediatric patients limited the power to detect subtle age-based differences in the effects of hydrocephalus on mortality. This limitation is important to consider when applying the results to clinical practice, particularly in pediatric populations.

Multicenter prospective studies are needed to validate these findings and assess whether standardized hydrocephalus management across age groups may improve survival across the lifespan.

## Conclusions

Hydrocephalus is a strong predictor of in-hospital mortality in TBM. We found no statistically clear evidence that age modified the association between hydrocephalus and mortality across the lifespan. However, this finding should be interpreted cautiously because the pediatric subgroup was relatively small and the confidence intervals were wider. These findings support early detection and timely management of hydrocephalus in TBM patients of all ages, while emphasizing that age remains clinically relevant for absolute mortality risk stratification.

## Data Availability

The original contributions presented in the study are included in the article/supplementary material, further inquiries can be directed to the corresponding authors.
